# Expanding the PURA syndrome phenotype with manifestations in a Japanese female patient

**DOI:** 10.1038/s41439-022-00189-7

**Published:** 2022-04-19

**Authors:** Yuya Fukuda, Yoshimasa Kudo, Makoto Saito, Tadashi Kaname, Tohru Oota, Reikichi Shoji

**Affiliations:** 1 Department of Pediatrics, Steel Memorial Muroran Hospital, Hokkaido, Japan; 2 Department of Genome Medicine, National Center for Child Health and Development, Tokyo, Japan; 3 Research Institute of Health Sciences, Health Sciences University of Hokkaido, Hokkaido, Japan

**Keywords:** Paediatric neurological disorders, Epilepsy, Growth disorders

## Abstract

We report on a 15-year-old Japanese female patient with hypotonia and global developmental delay from the neonatal period who was revealed to carry a known pathogenic *PURA* variant (NM_005859.5:c.697_699del, p.Phe233del) by whole-exome sequencing. She had previously unreported clinical features, including a rectovestibular fistula, extremely short stature, and underweight, expanding the known phenotype of PURA syndrome.

Purine-rich element-binding protein A (PURA) syndrome (MIM #600473) is a rare genetic disorder characterized by moderate-to-severe intellectual disability with hypotonia, hypothermia, hypersomnolence, feeding difficulties, excessive hiccups, recurrent central and obstructive apnea, epileptic seizures, abnormal nonepileptic movements, and abnormal vision^1^. Purine-rich element-binding protein (Pur) is a sequence-specific, single-stranded, nucleic acid-binding protein that is highly conserved from bacteria to humans^2^. Purα is a Pur member involved in neuronal proliferation, dendrite maturation, and the transport of mRNA to translation sites in hippocampal neurons; it also has an important role in postnatal brain development^3,4^.

According to the PURA Syndrome Foundation (https://www.purasyndrome.org/family), over 478 diagnosed cases have been reported worldwide as of September 2021. However, because the disease is not widely known among clinicians, it is conceivable that many patients have yet not been diagnosed. Here, we report a Japanese female patient with hypotonia and global developmental delay carrying a known pathogenic *PURA* variant (NM_005859.5:c.697_699del, p.Phe233del). This patient had clinical features previously unreported in association with PURA syndrome, including underweight, an extremely short stature, and a rectovestibular fistula^5–9^.

The 15-year-old patient was first referred to our hospital at the age of 5 years. She is the third daughter of nonconsanguineous healthy parents, with a healthy older brother and sister. She was born at 37 weeks of gestation by vaginal delivery with a birth weight of 2740 g and Apgar scores of 7 and 7 at 1 and 5 min, respectively. She had labored breathing and hypotonia at birth and required oxygen therapy for several days. She also had poor sucking and weight gain and needed nasogastric tube feeding for 2 months. Brain computed tomography, magnetic resonance imaging (MRI), newborn screening tests for congenital metabolic diseases, and blood tests performed during hospitalization were all normal. Karyotype analysis showed a normal female karyotype of 46,XX. A chromosome 15 methylation test for Prader–Willi syndrome was normal, and there were no *SMN1* gene mutations. She was discharged at 3 months of age with a weight of 4734 g. At the age of 1.5 years, she weighed 8.7 kg (–1.2 SD). She had severe allergies to eggs, milk, and wheat and experienced anaphylaxis several times. She also had severe constipation during infancy.

At the age of 5 years, she presented with marked hypotonia, a myopathic face, frontal bossing, a high-arched palate, esotropia, almond-shaped palpebral fissures, long, thin fingers, and soft skin (Fig. 1A). At the age of 7 years, she developed atonic, tonic, and gelastic seizures. An electroencephalogram (EEG) revealed occasional spikes, mainly over the left parietal region (Fig. 1B). She was diagnosed with epilepsy and was prescribed several anti-seizure medications. A nonepileptic exaggerated startle response to slight stimulation involving splaying of her arms was also often seen. Repeat brain MRI revealed no abnormalities.

**Fig. 1:**
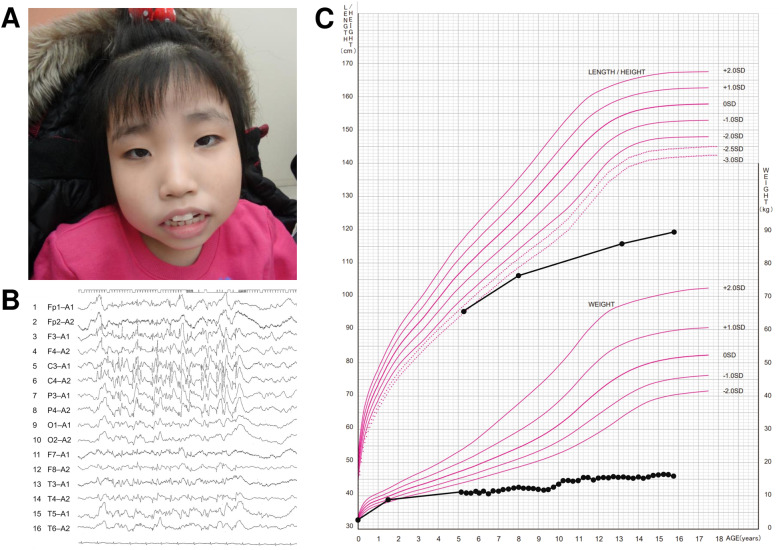
Clinical findings. **A** The patient at 13 years of age showing a myopathic face, frontal bossing, a high-arched palate, esotropia, and almond-shaped palpebral fissures. Written informed consent for the publication of photographs was obtained from the patient’s parents. **B** EEG of the patient performed at 8 years of age. **C** Growth chart of the patient showing height and weight. Growth curves are based on a cross-sectional growth chart for Japanese girls.

At the age of 10 years, feces was observed to pass from her vaginal vestibule. Physical examination revealed a slight fistula in her vaginal vestibule but no imperforate anus. An enema of gastrografin containing pyoctanin showed no fistula from her rectum to vaginal vestibule, but pyoctanin was attached to the vaginal vestibule after examination. Histological examination of the excrement from her vaginal vestibule showed the presence of food residue, so we diagnosed her with a rectovestibular fistula.

The patient has global developmental delay; she achieved head control at 6 years of age, sat alone at 7 years of age, and stood with support at 10 years of age. At 15 years of age, she cannot stand alone and needs full assistance when bathing and at mealtimes. Mobility is only possible in a wheelchair operated by another individual. She demonstrates social smiling and can use jargon but does not speak recognizable words. Growth was also significantly delayed; her height was 106 cm (–3.5 SD), her weight was 12 kg, her body mass index (BMI) was 10.7 at 8 years of age, and at 15 years of age, her height is 118 cm (–7.6 SD), her weight is 16 kg, and her BMI is 11.5 (Fig. 1C).

To confirm a molecular diagnosis, we analyzed DNA samples of the patient obtained at the age of 11 years and of both parents by whole-exome sequencing (WES) using the HiSeq 2500 System (Illumina, San Diego, CA). DNA samples were extracted from peripheral blood after receiving written informed consent from her parents. WES detected a heterozygous in-frame deletion (NM_005859.5:c.697_699del, p.Phe233del) in exon 1 of *PURA* in the patient but not her parents. This was confirmed by Sanger sequencing.

*PURA* p.(Phe233del) is the most commonly reported variation and was first identified by Hunt et al. in 2014; our patient is the ninth reported to carry the variant^5–9^. Table 1 compares all nine patients with the variant, revealing common clinical features including neonatal hypotonia, postnatal hypotonia, intellectual disability, language delay, and motor development delay. However, Patient 14, described by Reijnders et al., first sat alone at 1–2 years of age, while our patient achieved this at 7 years of age. Similarly, patient DB15-027, described by Lee et al., was able to walk alone at 4 years of age, while our patient remains unable to do so at 15 years of age. This suggests the presence of a wide range of developmental phenotypes among patients with the same *PURA* variant. Feeding difficulties, respiratory problems, hypersomnolence and hypothermia in the neonatal period, an exaggerated startle response, epilepsy, EEG abnormalities, brain MRI abnormalities, gastrointestinal abnormalities (e.g., constipation or drooling), vitamin D deficiency, and dermatological abnormalities (e.g., soft skin and cutis laxa) were observed in more than half of the nine patients. Our patient shared these clinical features, except for hypersomnolence and hypothermia in the neonatal period, brain MRI abnormalities, and vitamin D deficiency. The clinical features of patients carrying the *PURA* p.Phe233del variant do not differ from those reported for all patients with PURA syndrome, suggesting that it is difficult to describe reliable genotype–phenotype correlations^5,9^.

**Table 1:** Clinical features of patients with *PURA* p.Phe233del variant.

Clinical features	This patient	Hunt et al.^6^	Tanaka et al.^7^	Reijnders et al.^5^			Lee et al.^8^		Cinquina et al.^9^
		Patient 4	Patient 4	Patient 4	Patient 5	Patient 14	DB15-027	DB16-032	
Background									
Reported age	15 years	6 years, 9 months	6 months	14 years	19 years	9 years	4 years	13 months	3 years
Sex	Female	Female	Female	Male	Female	Male	Male	Female	Female
Inheritance	AD–de novo	AD–de novo	N/A	de novo	de novo	AD–de novo	N/A	N/A	de novo
Gestation									
Delivery	Vaginal delivery	Selective caesarean section	N/A	Vaginal delivery	Vaginal delivery	Vaginal delivery	N/A	N/A	Vaginal delivery
Gestational age	37 weeks	38 weeks	N/A	42 weeks	42 weeks	42 weeks	N/A	N/A	41 weeks
Birth weight	2740 g	3012 g	N/A	N/A	3660 g	3629 g	N/A	N/A	3300 g
Neonatal problems									
Hypotonia	Yes	Yes	Yes	Yes	Yes	Yes	Yes	Yes	Yes
Feeding difficulties	Yes	Yes	N/A	Yes	Yes	No	Yes	Yes	Yes
Requiring tube feeding	Yes (since 2 months of life)	Yes	N/A	No	No	–	No	Yes (since 5 weeks of life)	Yes (since 2 months of life)
Respiratory problems	Yes	Yes	N/A	Yes	Yes	No	No	Yes	Yes
Apnea	Yes	Yes	N/A	Yes	Yes	No	No	Yes	No
Hypersomnolence	No	N/A	N/A	Yes	Yes	N/A	N/A	N/A	No
Hypothermia	No	Yes	N/A	N/A	Yes	N/A	N/A	N/A	No
Development									
Intellectual disability	Yes	Yes	Yes	Yes	Yes	Yes	Yes	Yes	Yes
Language delay	Yes	Yes	N/A	Yes	Yes	Yes	N/A	N/A	Yes
Gait	Not achieved	Not achieved	Not achieved	Broad based	Broad based with support	Unsteady	Ambulatory	Not achieved	Not achieved
Age of first step	Not achieved	Not achieved	Not achieved	N/A	4 years, but regression since onset of seizures	7 years	N/A	Not achieved	Not achieved
Age of sitting unsupported	7 years	Not achieved	N/A	N/A	15 months	1–2 years	N/A	N/A	3 years
Growth									
Height	118 cm (−7.6 SD)	123 cm (75%tile)	N/A	147.3 cm (−1.55 SD)	164 cm (−1.0 SD)	121 cm (−2SD)	N/A	N/A	N/A
Weight	16 kg	25 kg (75%tile)	N/A	37.2 kg (0.38 SD)	48 kg (−0.5 SD)	32 kg (+0.5 SD)	N/A	N/A	N/A
BMI	11.5	16.5	–	17.1	17.8	21.9	–	–	–
Neurological abnormalities									
Postnatal hypotonia	Yes	Yes	Yes	Yes	Yes	Yes	Yes	Yes	Yes
Exaggerated startle response	Yes	N/A	N/A	No	Yes	Yes	No	No	Yes
Epilepsy	Yes	Yes	No	No	Yes	Yes	No	Yes	No
Age of onset	7 years	14 months	–	–	2-3 years	3 years	–	2 weeks	–
EEG abnoromalities	Yes	Yes	No	No	Yes	Yes	N/A	N/A	No
Brain MRI abnormalities	No	Delayed myelination, excessive extraaxial fluid spaces	Periventricular leukomalacia	Delayed myelination	Delayed myelination	Delayed myelination	Thin white matter, excessive extraaxial fluid spaces	N/A	No
Other abnormalities									
Cardiovascular	No	N/A	N/A	ASD	No	VSD, aberrant left subclavian artery	No	VSD	No
Respiratory	No	N/A	N/A	Apneas > age 1 year	Apneas > age 1 year	No	No	No	No
Gastrointestinal	Constipation, drooling, rectovestibular fistula	N/A	Swallow problems	No	Constipation, drooling	Drooling	N/A	Constipation	Drooling
Opthalmologic	Strabismus	Cortical visual imparment	Cortical visual imparment	Refraction abnormality, strabismus	Nystagmus, strabismus, cortical visual imparment	Refraction abnormality, strabismus	Cortical visual imparment	No	No
Endocirine									
Vitamin D deficiency	No	Yes	N/A	Yes	Yes	Yes	N/A	N/A	No
Dermatological	Soft skin	N/A	N/A	No	Soft skin	No	N/A	N/A	Cutis laxa
Skeletal	Scoliosis	N/A	N/A	No	Scoliosis, hip dysplasia	No	No	No	No
Urogenital	No	N/A	N/A	No	No	No	N/A	N/A	No

*AD* autosomal dominant, *ASD* atrial septal defect, *BMI* body mass index, *EEG* electroencephalogram, *MRI* magnetic resonance imaging, *N/A* not applicable, *SD* standard deviation, *VSD* ventricular septal defect.

Our patient had a rectovestibular fistula, which has not previously been reported in patients with PURA syndrome. Congenital rectovestibular fistulas comprise the majority of anorectal malformations^10^. However, female anorectal malformations can be difficult to diagnose precisely because of anatomical complexities and the approximation of genital organs^11^. Acquired rectovestibular fistulas are often a symptom of a disorder such as Crohn’s disease, pelvic infection, and malignancy^12^. Our patient was diagnosed with a rectovestibular fistula at the age of 10 years when feces were observed to pass from her vaginal vestibule, but it was difficult to distinguish whether this was congenital or acquired by physical examination and imaging. Therefore, although it is unclear whether the fistula is associated with the *PURA* variant, our patient may expand the phenotypic spectrum associated with PURA syndrome.

Patients with PURA syndrome often have a short stature^5,13^. Indeed, Reijnders et al. reported that the height of 8/49 (16%) patients was ≤2.5 SD^5^. However, the extent of short stature and low weight of our patient, as shown in Fig. 1C, is incomparably stronger than in previous reports. Our patient lacks the limb shortening characteristic of achondroplasia, which is associated with a markedly short stature. Severely stunted height and weight growth can also result from inadequate caloric intake (such as that caused by difficulties with nursing or limited food availability), inadequate caloric absorption (such as that resulting from metabolic or gastrointestinal disorders), or excessive caloric expenditure/ineffective utilization (e.g., resulting from hyperthyroidism, diabetes, pulmonary, or cardiac conditions)^14^. Our patient could eat a certain amount of food with assistance, and physical examinations revealed no metabolic disorders, hyperthyroidism, diabetes, or cardiac disease. The possibility of inadequate caloric absorption from gastrointestinal disorders cannot be ruled out, but it should be noted that PURA syndrome itself can strongly slow growth.

In conclusion, we report on a 15-year-old Japanese female with hypotonia and global developmental delay from the neonatal period that had an unknown cause for many years but was finally revealed to result from the *PURA* p.Phe233del variant, which was identified by WES. In a comparison with eight previously reported patients carrying the same variant, all shared neonatal/postnatal hypotonia, intellectual disability, and language delay. However, other phenotypes vary widely among patients, suggesting that it is difficult to identify reliable genotype–phenotype correlations for PURA syndrome. Our patient also has a rectovestibular fistula and is of a markedly short stature and low weight; these findings may expand our current knowledge of the phenotypic spectrum associated with PURA syndrome.

## Data Availability

The relevant data from this Data Report are hosted at the Human Genome Variation Database at 10.6084/m9.figshare.hgv.3155.

## References

[1] Reijnders M. R. F. et al. PURA-related neurodevelopmental disorders. In *GeneReviews® [Internet]*. (eds Adam, M. P. et al.) (University of Washington, 2017). 28448108

[2] Johnson EM, Daniel DC, Gordon J (2013). The Pur protein family: genetic and structural features in development and disease. J. Cell Physiol..

[3] Khalili K (2003). Purα is essential for postnatal brain development and developmentally coupled cellular proliferation as revealed by genetic inactivation in the mouse. Mol. Cell Biol..

[4] Hokkanen S (2012). Lack of Pur-alpha alters postnatal brain development and causes megalencephaly. Hum. Mol. Genet..

[5] Reijnders MRF (2018). PURA syndrome: clinical delineation and genotype-phenotype study in 32 individuals with review of published literature. J. Med. Genet..

[6] Hunt D (2014). Whole exome sequencing in family trios reveals de novo mutations in PURA as a cause of severe neurodevelopmental delay and learning disability. J. Med. Genet..

[7] Tanaka AJ (2015). De novo mutations in PURA are associated with hypotonia and developmental delay. Cold Spring Harb. Mol. Case Stud..

[8] Lee BH (2018). Expanding the neurodevelopmental phenotype of PURA syndrome. Am. J. Med. Genet. A..

[9] Cinquina V (2021). Expanding the PURA syndrome phenotype: a child with the recurrent PURA p.(Phe233del) pathogenic variant showing similarities with cutis laxa. Mol. Genet. Genom. Med..

[10] Choudhury SR (2017). Anorectal agenesis with rectovaginal fistula: a rare/regional variant. J. Indian Assoc. Pediatr. Surg..

[11] Holschneider A (2005). Preliminary report on the international conference for the development of standards for the treatment of anorectal malformations. J. Pediatr. Surg..

[12] Champagne BJ, McGee MF (2010). Rectovaginal fistula. Surg. Clin. North Am..

[13] Boczek, N. J. et al. Expansion of PURA-related phenotypes and discovery of a novel PURA variant: a case report. *Child Neurol. Open*. **7**, 2329048X20955003 (2020). 10.1177/2329048X20955003 PMC7573717 33117858

[14] Grissom M (2013). Disorders of childhood growth and development: failure to thrive versus short stature. FP Essent..

